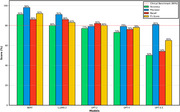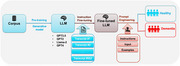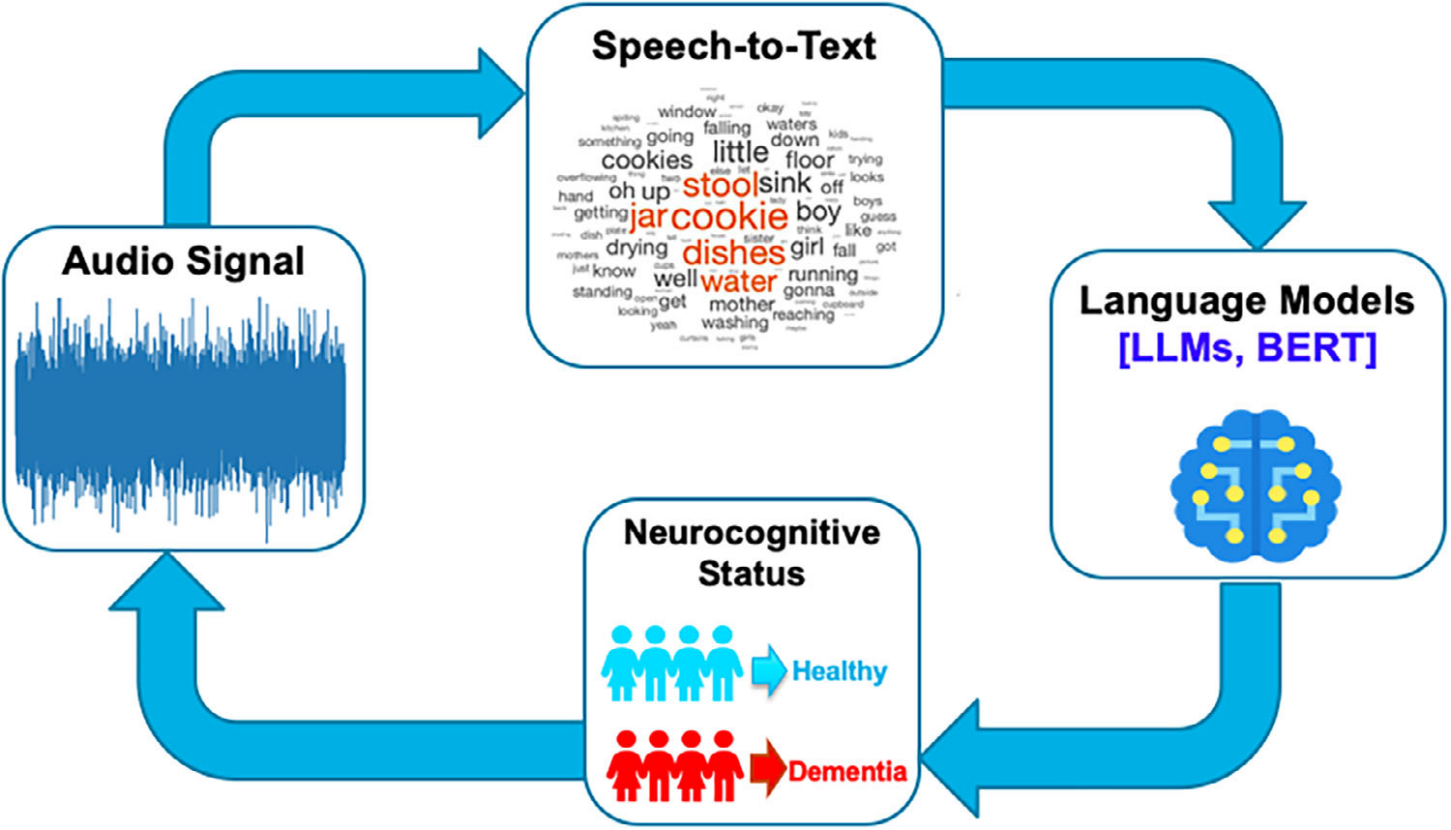# Automated Detection of Early‐Stage Dementia Using Large Language Models: A Comparative Study on Narrative Speech

**DOI:** 10.1002/alz70861_108503

**Published:** 2025-12-23

**Authors:** Kevin Mekulu, Faisal Aqlan, Hui Yang

**Affiliations:** ^1^ Pennsylvania State University, University Park, PA USA; ^2^ University of Louisville, Louisville, KY USA

## Abstract

**Background:**

Early detection of dementia is essential for timely intervention, yet current methods often rely on subjective assessments. Advances in natural language processing (NLP) offer new opportunities for objective and scalable screening. This study compares the performance of two classes of language models for dementia detection from narrative speech: pre‐trained encoder‐based models and autoregressive large language models (LLMs).

**Method:**

Using the DementiaBank Pitt Corpus, we evaluated BERT as a representative pre‐trained language model (PLM) and compared it to autoregressive LLMs including GPT‐2, GPT‐3.5 Turbo, GPT‐4, and LLaMA‐2. Although all models are pretrained, we use "PLM" to refer to encoder‐based architectures like BERT, and "LLM" to refer to large, decoder‐based models trained for generative tasks. Transcripts from the Boston Cookie Theft picture description task were used to classify dementia versus control participants. Models were assessed using sensitivity and specificity

**Result:**

BERT achieved the highest classification performance with 86% sensitivity and 95% specificity. LLaMA‐2 followed with 86% sensitivity and 89% specificity, while GPT‐4 reached 76% sensitivity and 82% specificity. While encoder‐based PLMs outperformed in structured classification tasks, LLMs showed complementary strengths in modeling narrative richness and subtle linguistic cues.

**Conclusion:**

BERT achieved the highest classification performance with 86% sensitivity and 95% specificity. LLaMA‐2 followed with 86% sensitivity and 89% specificity, while GPT‐4 reached 76% sensitivity and 82% specificity. While encoder‐based PLMs outperformed in structured classification tasks, LLMs showed complementary strengths in modeling narrative richness and subtle linguistic cues.